# The Relation of Pathology to the Practice of Medicine and Surgery*Read before the Alumni of Medical Department, Niagara University, Buffalo, N. Y., April 10, 1888.

**Published:** 1888-06

**Authors:** Frank W. Ross

**Affiliations:** Elmira, N. Y.


### *THE RELATION OF PATHOLOGY TO THE PRACTICE OF MEDICINE AND SURGERY.**

By FRANK W. ROSS, M. D., Elmira, N. Y.

I believe that few physicians and students properly appreciate the value of the study of pathology and its relations to medicine and surgery.

It is the great basis upon which we must work, and without which we are at sea.

The *dry details* of pathological anatomy and pathological processes are not, as a rule, very inviting or entertaining to the student, but the more he enters into practice, the more he sees of disease, and the greater his reverence for this practical line of study in all its departments.

I do not mean simply pathology as studied in the dead-house alone. Pathological anatomy, gross and microscopical, is merely a record and *prima facie* evidence of *what has been done*, but the anatomical part, combined with a knowledge of the *various processes* which bring about the condition, is true pathology.

In "Quain's Dictionary of Medicine," pathology is defined as the "science of disease."

I believe that pathology is the foundation, the basis, for the rational treatment of disease, both medical and surgical.

No one can hope to treat a disease in a rational and scientific manner without a knowledge of its pathology.

To know the changes which the various organs of the body undergo, and the constant pathological lesion of the known disease, is the scientific part of medicine, and is in itself an exact science.

The administration of medicine is, from the very nature of the conditions which surround it, in the main part empirical and experimental.

Here we have not only the changed condition of the body, the abnormal actions of the organs, and the faulty assimilation of a remedy which modifies to such a great extent the action, but

* Read before the Alumni of Medical Department, Niagara University, Buffalo, N. Y., April 10, 1888.

we have also to deal with the *drugs* themselves, which too often are impure, adulterated, or deteriorate from age or other causes, to say nothing of the dishonesty or meddlesome interference of the unscrupulous pharmacist.

All of these have a tendency to place the science of medicine in an unenviable position before the laymen, who believe that every dose of medicine given is an experiment, and consider themselves *too* often to have been the victim of *misplaced* confidence and powerful drugs!

To these they ascribe the unfortunate results or sequels of the disease through which they have just passed, forgetting, or not knowing, that disease in itself *always* leaves its mark upon the body of its victims in some form.

This result, in all degrees of intensity, *is the constant pathological lesion*, and the study of the various processes which produce it, and of the changes of the organs, as seen by the unaided eye and microscope, is the scientific pathology of to-day.

But few physicians, prior to the last decade, were versed in this art, and the present knowledge is the accumulation of the past, and disease is now studied from this, a pathological standpoint: more reliance is placed upon it, the diagnosis, the mapping out of a line of treatment, the prognosis, *all* are shaped from the *pathological lesion*, which is known to exist.

In fact, when we once know the pathology of a disease, we have made advances which eclipse and surpass in every way the experiments and empiricisms of ages.

This disease is isolated from its fellows, and its care and treatment becomes an exact science.

No matter what the result of an investigation may be in a given disease, it may show that a hitherto-supposed incurable disease is curable, or that a supposed curable diseases is not curable.

It satisfies the demand of exact science, and it places us upon a firm basis, and mankind is the gainer thereby.

As a result of careful study, the changes in the treatment of fever are simply marvelous.

We have all been told of the old methods of the treatment of fevers, in particular the bleeding, the purging, and the tartar emetic, "just enough to keep the stomach a little sick," the withdrawing of water and of nourishment. They seem a nightmare!

Thanks to our knowledge of the pathology of fever, these things have undergone a revolution. It is no longer the rule to bleed, physic, salivate, nauseate, and starve our patients, but from an exact knowledge of the pathology, the entire line of treatment is changed.

The first thing which is pressed upon the student's mind is the nourishment, the administration of food and drink in proper quantities.

You are taught that *disease itself* is, as a rule, *a depressant* of the vital forces, which must be counteracted by rest, food and, *perhaps*, *medicine*.

You learn that disease is not *cured* by medicine, but that the *vis medicatrix naturæ* is the all-healing power before which we must bend, towards the maintaining the system and *assisting* nature, our mission is accomplished; few diseases, *if any*, succumb to the simple *vis medicatrix alone!*

You are, to progress in your art, not only obliged to listen to eloquent and instructive lectures, and to read learned and exhaustive treatises, but to stand at the bedside of your patients and watch the signs and symptoms of disease, and when death ensues, you must follow the body to the dead-house, and there study both the gross and microscopical lesions, which are eloquent reminders of the *real causes of* disease, its *symptoms* and course, and an explanation of the fatal termination, it says plainly to us, in the matter of healing art, "thus far canst thou go, and no farther."

It tells you that disease, no matter how simple, is a hydra-headed monster with which we have to cope, and *what* the limit of *restoration to health is* that most so-called cures are merely "a stay in proceedings," and nothing more.

I well remember the first pathological specimen of the ulcerated pyers patches of typhoid fever which came under my eye.

It seemed *impossible* to expect any result by *purely medicinal treatment* of the diarrhœa which resulted. Not long after, a young man, who knew more of medicine than of disease, was asked, as a quiz, his treatment of the diarrhœa in the ulcerative stage of typhoid fever. Said he: "I would use three grains of the sub-nit. bismuth every three or four hours, and check it; the bismuth acting as an astringent upon the ulcers." Whereupon the quiz-master said: "Just imagine that large dose of three grains of bismuth wandering up and down *thirty feet of gut*, saying: 'Whare's that ulcer, whare's that ulcer?'"

The present treatment of those diseases, which have no known constant pathological lesion also in those diseases where there exists a doubt as to the pathology, is unsatisfactory, and must, of necessity, be empirical.

The diversity of opinion of so-called experts in medicine and surgery, and the wide variations of, and seemingly diametrically opposed, methods of treatment of any disease, where the pathology is not known, calls for the condemnation and criticism of people who say that if *we differ* so widely in these respects, there is *no foundation* or basis to medicine, and we are consequently unjustly censured for *not knowing what* no power on earth has yet been able to disclose.

As soon as the pathology is understood, the battle ceases, and a new field is investigated. In this manner, medical science, slowly fighting every inch of ground, while disease stubbornly refuses an advance into her domain, the pathology of disease is gradually unfolding to the ever-watchful and vigilant eye of science, her hidden mysteries and wonderful power for destruction of mankind; the fight is stubborn, uncompromising, and, in some diseases, *days*, *months*, and even *ages*, fail to reveal the *truth*.

We are just as much in the dark to-day, as to the cause and pathology of *diabetes*, as we were a century ago.

We know that there is a condition of the system, in which abnormal quantities of sugar are present in the urine, and that, as a rule, this disease is progressive and fatal; yet it has no *constant pathological lesion known;* whether it is a disease of the

*nerves*, *brain*, or the *liver*, *stomach* or *kidneys*, we are not certain. In some cases, it seems to be one cause; in others, an apparently different state of affairs exists. Hence, we have to-day no rational treatment for this disease which we call diabetes. We only know its constant symptom, but little or nothing of the *disease* itself.

On the other hand, we know so well the pathology of such diseases as typhoid fever and pneumonia, and their constant pathological lesions, that we can tell almost to the hour the changes which the organs are undergoing at a given stage of the disease.

Knowing this, *we have the key* which unlocks the mystery, and which serves as a guide for our *care and treatment*. *Knowing them*, we are prepared to stand on guard, to watch, with jealous and vigilant eye, all the symptoms, and rightly interpret their meaning.

In these diseases, we have reached the acme of perfection in the healing art of the present age, because we have our beacon-light ahead, while we hold the rudder to steer the craft, between Scylla and Charybdis. Although there may be hidden rocks ahead, we are as safe under this guidance as mortal can hope to be. Only the Omnipotent can tell that which is beyond our ken.

To be skilled in the art of medicine and surgery, requires that a physician should know something outside of the action and administration of drugs, or the use of the knife. He must be skilled in the *exact sciences*—pathology, anatomy, chemistry, and physics, etc.—else he is no more a scientific physician than the ignorant quack who promises to cure all the diseases to which the flesh is heir. It is a lack of the proper scientific basis of a medical education, and a failure to study and understand pathology, which leads men otherwise versed in medicine to expect miraculous and impossible cures of known pathological lesions.

The *pathologist* is a *safer man to treat disease* than an *empiric*, yet we often see the *ultra-pathologist*, who considers disease *only* from the *dead-house* standpoint, a signal failure when he attempts to cope with disease, or utterly skeptical as to the efficacy of any remedial agent.

He is apt to undervalue the wonderful restorative power of nature, and forgets that most of the organs of the body are capable of doing more work than is often required of them, and can also assist, for a variable length of time, another organ whose functions may be crippled or suppressed.

This allows the diseased member rest. Notably, the bowels may assist the kidneys, and, by increased activity, carry off the effete material which the kidneys should remove. The kidneys may act for the liver, or the skin for the kidneys, etc.

Thus, by all the organs working in harmony, assisting each other, we are able to pass crises which would otherwise cause a fatal result.

The rectum may be used to supplement the action of the stomach, while all the special senses are aided one by the other. Again, we know of pathological changes producing others to compensate for loss or change of function. Notably, in diseases of the *heart* and *circulatory system*, we have *cardiac hypertrophy* to overcome the resistance offered by a disease of the arteries, which requires more force to carry on the circulation. Notably, in organic diseases of the valves, where not only hypertrophy *is compensatory*, but an *actual lesion of the valves* will ensue to assist the left heart; for example, a tricuspid regurgitation, when not congenial, is almost always compensatory to a nitral lesion ot the left heart.

In the domain of surgery, the study of tumors has been a great boon, and we now know, by the examination of a tumor, whether it is malignant or not, and often base our diagnosis on the microscopical appearance of a few cells.

In the case of the Emperor of Germany's throat, much reflection has been cast on the opinion of expert pathologists, but we must remember that Germany is not America; there, pathology and science must work in harmony with the "will of the Sovereign." The Crown Prince played for a *great stake*. Had his disease been pronounced cancer, he could not, by the laws of the land, be Emperor, which means $100,000 a year, and to his wife the *title of Empress*.

This will readily explain why we know so little definitely of his case, and why Queen Victoria sent McKenzie to care for the Crown Prince; it *was worth something for her daughter to be the Empress Dowager.* The exact truth of the case may never be known until the death of the Emperor.

The pathology of embolism, from endocarditis, cardiac vegetation, from a thrombus of a vein, with perhaps a resulting paralysis, is easily explained, more readily understood, cared for, and fatal issues often averted.

The pathology of *involution of the uterus* is in itself the guide for the treatment of the lying-in woman.

The arrangement of cells and distribution of blood vessels explain to the merest tyro why *sarcoma* is so apt to be *multiple* and almost always recurrent, while cancer usually advances by *progressing* itself through all the tissues, and rarely in distant parts of the body, except in advanced and ulcerated stages, which places it in the same condition as sarcoma, for in cancer we find the blood-vessels in the stroma; in sarcoma they ramify *among* the cells, offering the best possible means of transportation to various parts of the body.

Thus it is with most diseases we have a ready and rational explanation of the leading symptoms from the pathology.

Our facilities for the study of pathology in general practice are not large, yet if we *make the most of all* the cases that come before us, hold post-mortems whenever we can, we cannot but learn more or less of pathology, even in isolated places.

I have heard the great Marion Sims remark that many of his investigations and original researches were made in a small hospital in his southern home, while he was comparatively a young man, and his future and wider experience only verifies his previous investigations.

If you know all there is to the pathology of one case of typhoid fever, you know entire pathology. One by one, you can study and investigate the pathology and conditions of various diseases, and thus place yourself on a sound basis with which to practice *medicine and surgery.*

